# Methotrexate sensitizes drug-resistant metastatic melanoma cells to *BRAF* V600E inhibitors dabrafenib and encorafenib

**DOI:** 10.18632/oncotarget.24341

**Published:** 2018-01-29

**Authors:** Kayleigh C. Ross, Kevin F. Chin, Daehwan Kim, Christopher D. Marion, Timothy J. Yen, Vikram Bhattacharjee

**Affiliations:** ^1^ Evol Science, Philadelphia, PA 19104, USA; ^2^ Fox Chase Cancer Center, Philadelphia, PA 19111, USA

**Keywords:** dabrafenib, encorafenib, methotrexate, metastatic melanoma, pancreatic cancer

## Abstract

Acquired resistance of metastatic melanoma (MM) tumors to *BRAF* V600E inhibitors (BRAFi’s) is commonplace in the clinic. Habitual relapse of patients contributes to <20% 5-year survival rates in MM. We previously identified serine synthesis as a critical detrminant of late-stage cancer cell resistance to BRAFi’s. Pre-treatment with DNA damaging agent gemcitabine (a nucleoside analog) re-sensitized drug-resistant cancer cells to BRAFi’s dabrafenib and vemurafenib. Importantly, the combination treatments were effective against BRAF wild type cancer cells potentially expanding the clinical reach of BRAFi’s. In this study, we identify the antifolate methotrexate (MTX) as a sensitizer of acquired- and intrinsically-resistant MM cells to BRAFi’s dabrafenib and encorafenib. We identify a novel, positive correlation between dabrafenib treatments and repair delay of MTX induced single-strand DNA (ssDNA) breaks. Cells arrest in G1 phase following simultaneous MTX + dabrafenib treatments and eventually die via apoptosis. Importantly, we identify RAS codon 12 activating mutations as prognostic markers for MTX + BRAFi treatment efficacy. We describe a method of killing drug-resistant MM cells that if translated has the potential to improve MM patient survival.

## INTRODUCTION

In this study, we describe experiments that exploit natural cell proliferative mechanisms of metastatic melanoma (MM) drug-resistance to sensitize otherwise resistant cancer cells to unique combination therapies. We show that we can kill melanoma cells by simultaneously activating DNA damage checkpoints with a DNA damaging agent and cell proliferative signaling via hyperactivation of the Mitogen-activated protein kinase (MAPK) cascade with BRAF (v-Raf murine sarcoma viral oncogene homolog B) inhibitors (BRAFi’s). We have previously reported this unique and novel method of cell killing [1] that is potentially broad reaching among late-stage solid tumor cancers independent of specific disease states because it is effective regardless of BRAF mutational status. Exploiting this combination to successfully kill otherwise resistant cancer cells may hold the potential to significantly improve and extend the clinical efficacy of BRAFi’s.

MM is expected to claim over 10,000 deaths in the US this year (cancer.net). MM is classified as the deadliest type of skin cancer. As a late stage cancer, MM disproportionately affects men over women by a ratio of 1.5 to 1. The 5-year survival rate of patients diagnosed with MM is <20% (American Cancer Society, 2017). Though advances in selective chemotherapeutic and immunotherapy regimens have improved short-term patient health, extending lives, intrinsic and acquired drug resistance has become a major hindrance in the clinic contributing to an overall low 5-year survival in MM [2]. Therefore, there is a need for identifying effective therapeutic regimens specifically targeting acquired drug-resistant MM cells.

BRAF is a serine/threonine kinase that is mutated in ∼50% of cutaneous melanoma clinical samples [2]. The most common mutation found is a valine (V) to glutamic acid (E) substitution at codon 600 [3, 4]. The oncogenic V600E mutation causes hyperactivation of BRAF kinase activity and results in hyperinduction of the MAPK cascades. BRAFi’s that selectively inhibit *BRAF* V600E mutant gene product have received FDA approval for treatment of unresectable MM. Dabrafenib, which received FDA approval in 2013, disrupts *BRAF* V600E homodimerization thus preventing BRAF activation which in turn blocks downstream MAPK cascade activation [5]. However, in MM cells that express wild type (WT) BRAF, dabrafenib and related BRAFi’s are contraindicated because they allosterically stimulate BRAF kinase which leads to hyper-proliferation via the MAPK cascade activation [6, 7]. Thus, dabrafenib was approved specifically for treatment of MM that express the *BRAF* V600E mutant.

Initial responses to dabrafenib and related BRAFi vemurafenib were promising in the clinic. However, subsequent drug-acquired tumor resistance and patient relapse became commonplace [8]. Within 1 year of treatment, the clinical rates of acquired resistance to BRAFi’s dabrafenib and vemurafenib in MM stand at 33% and 45% respectively [9, 10]. Combination treatments with dabrafenib and MEK1/2 inhibitors have shown efficacy against *BRAF* V600E melanoma [11, 12], but acquired drug resistance also developed to these therapeutic combinations [13]. Recently, encorafenib (LGX818; BRAFi and inducer of senescence and autophagy [14]) and binimetinib (MEK1/2 inhibitor) combination treatments have been shown to be cytostatic and hold promise against BRAF V600E tumors in multiple disease states ([15, 16] and (NCT01909453)), but acquired resistance has developed to this combination as well [17]. Overall, the MAPK pathway has been a major therapeutic target in MM since the pathway is often hyperactivated during melanoma disease progression [18–21] and understanding and exploiting the biology of acquired drug resistance induced by downstream pathway proteins could potentially lead to positive outcomes in the clinic.

We previously reported serine synthesis as being critical to BRAFi resistance in MM *in vitro* [1]. The serine biosynthetic pathway contributes precursors to the folate cycle, which provides nucleotides for multiple DNA processes including DNA repair [22]. We showed that pretreating BRAFi resistant MM, pancreatic cancer, or non-small cell lung cancer cells with the nucleoside analog gemcitabine sensitized cells to dabrafenib and vemurafenib. Interestingly, in that study, methotrexate (MTX), an antifolate, treatment had an additive effect on the efficacy of gemcitabine + BRAFi treatments in a drug resistant cell line SK_MEL-28VR1.

In this study, we tested MTX as a sensitizer of dabrafenib in resistant MM cells. MTX is known to inhibit the folate cycle in melanoma cells [23] and is FDA approved for treatments of multiple cancers [24]. MTX is known to induce single strand breaks in cancer cells causing DNA damage checkpoint activation [25]. In 2D colony formation and 3D solid tumor spheroidal growth assays, we identify synergy between MTX and dabrafenib in acquired-resistant (SK-MEL28VR1) and intrinsically drug-resistant (501-mel) MM cells. Additionally, we show that MTX sensitized BRAF WT cells to encorafenib (LGX818), another BRAFi, in spheroidal growth assays. We also elucidate a novel dabrafenib induced DNA repair delay following MTX induced single strand DNA (ssDNA) breaks. Interestingly, DNA damage-induced arrest checkpoint is active and cells are arrested in G1 prior to cell death induction. Ultimately, we show that the MTX + dabrafenib combination treatment induces apoptosis and is cytotoxic to MM cells. Importantly, we identify a positive correlation between RAS codon 12 activating mutations and MTX+dabrafenib combination therapy efficacy. To our knowledge, we describe the first example of MTX-induced cytotoxic sensitization of drug-resistant cancer cells to dabrafenib or encorafenib. Importantly, we identify novel positive correlations between prolonged cell cycle arrest, DNA damage, MAPK hyperactivation, and apoptotic cell death following MTX + dabrafenib combination treatments.

## RESULTS

### Acquired drug-resistant SK-MEL-28VR1 and intrinsically drug-resistant 501-mel cells are sensitized to dabrafenib by MTX

**Figure 1:**
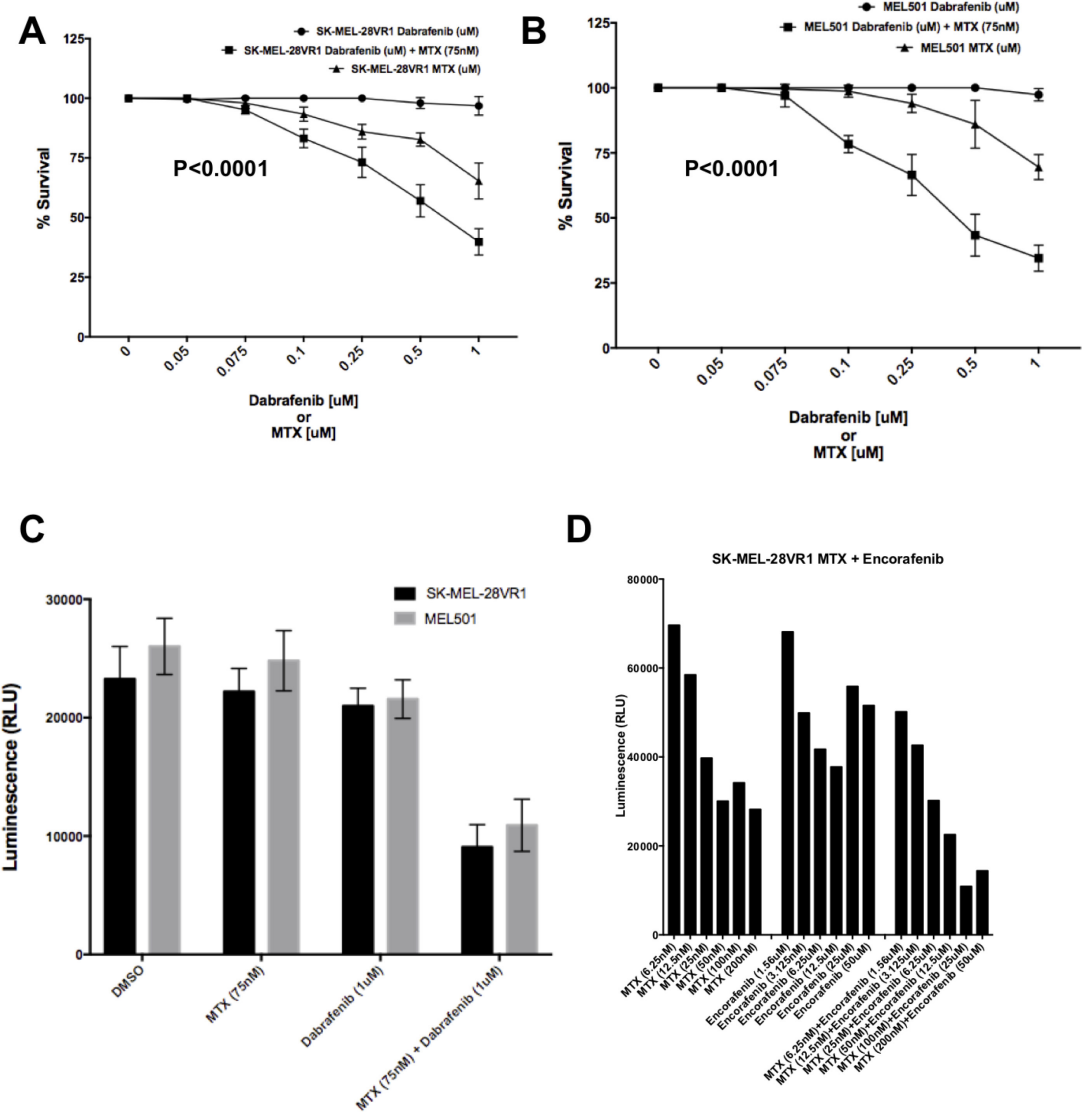
Sensitization of SK-MEL-28VR1 and 501-mel cells to BRAFi’s dabrafenib and encorafenib by MTX (**A**) Colony formation assays of SK-MEL-28VR1 cells following treatments with differential doses of MTX, dabrafenib, or MTX + dabrafenib (*n* = 3) (*p* < 0.0001). (**B**) Colony formation assays of 501-mel cells following treatments with differential doses of MTX, dabrafenib, or MTX + dabrafenib (*n* = 3) (*p* < 0.0001). (**C**) Spheroidal growth assays of SK-MEL-28VR1 and 501-mel cells following treatments with differential doses of MTX, dabrafenib, or MTX + dabrafenib (*n* = 3). (**D**) Spheroidal growth assays of SK-MEL-28VR1 cells following treatments with differential doses of MTX, encorafenib, or MTX + encorafenib.

10-day colony formation assays showed decreased cell survival of SK-MEL-28VR1 (Figure 1A) and 501-mel (Figure 1B) cells following MTX + dabrafenib double treatments compared to MTX or dabrafenib single treatments. SK-MEL-28VR1 cells (BRAFi acquired resistant variant derived from SK-MEL-28 MM line) are resistant to 1 µM of dabrafenib in 2D colony formation assays. The results clearly showed no difference in cell survival of SK-MEL-28VR1 cells treated with up to 1 µM of dabrafenib compared to vehicle alone (Figure 1A). However, when SK-MEL-28VR1 cells were treated with 75 nM of MTX, in addition to 0.25 µM, 0.5 µM, or 1 µM doses of dabrafenib, survival was reduced to 72%, 55%, and 42.5% relative to dabrafenib only treated cells. MTX single treatments at doses of 0.25 µM, 0.5 µM, or 1 µM, showed reduced viability but the MTX + dabrafenib combination exhibited higher cell killing. The difference between the combination curve and single dabrafenib curve was statistically significant (*p* < 0.0001).

Similarly, 501-mel cells were also sensitized to dabrafenib by MTX in colony formation assays (Figure 1B). 501-mel cells were intrinsically resistant to doses of dabrafenib up to 1 µM as determined in a 10-day colony formation assays. Addition of 75 nM MTX sensitized cells to a range of dabrafenib (0.1 µM, 0.25 µM, 0.5 µM, and 1 µM). Although 501-mel cell survival was reduced by MTX single treatments at doses of 0.25 µM, 0.5 µM, and 1 µM, they were more sensitive to MTX + dabrafenib combination treatment (Figure 1B). The difference between the combination curve and single dabrafenib curve was statistically significant (*p* < 0.0001).

Next, we tested the sensitivity of MM cells to the combination treatment in 3D solid tumor spheroidal growth assays. Both SK-MEL-28VR1 and 501-mel cells showed a reduction in cell growth when simultaneously treated with MTX and dabrafenib compared to MTX or dabrafenib single treatments or vehicle only treatments (Figure 1C). Viability of SK-MEL-28VR1 cells were found to be reduced by >50% with the combination treatment compared to single MTX, dabrafenib, or vehicle only treatments as quantitated by relative luminescence counts. Similarly, 501-mel cells also exhibited reduced viability following the combination treatments compared to single drug or vehicle treatments (Figure 1C). The 3D spheroidal growth assays extended the results obtained with the MTX + dabrafenib combination in the 2D colony formation assays in Figures 1A and 1B. Collectively, the 2D and 3D *in vitro* assays confirmed the efficacy of MTX as a sensitizer BRAFi resistant MM cells to dabrafenib. Additionally, we tested a second BRAFi, encorafenib, in combination with MTX in spheroidal growth assays (Figure 1D). Comparing single MTX or encorafenib treatments to MTX + combination treatments, the results clearly showed a decrease in cell survival following the combination treatments compared to single treatments with either drug. These results indicated that the increased efficacy of combination treatments was not a dabrafenib specific effect but a general effect common to multiple BRAFi’s.

### Dabrafenib activates the MAPK pathway and disrupts MTX induced single-strand DNA damage repair causing apoptosis in acquired resistant MM cells

**Figure 2:**
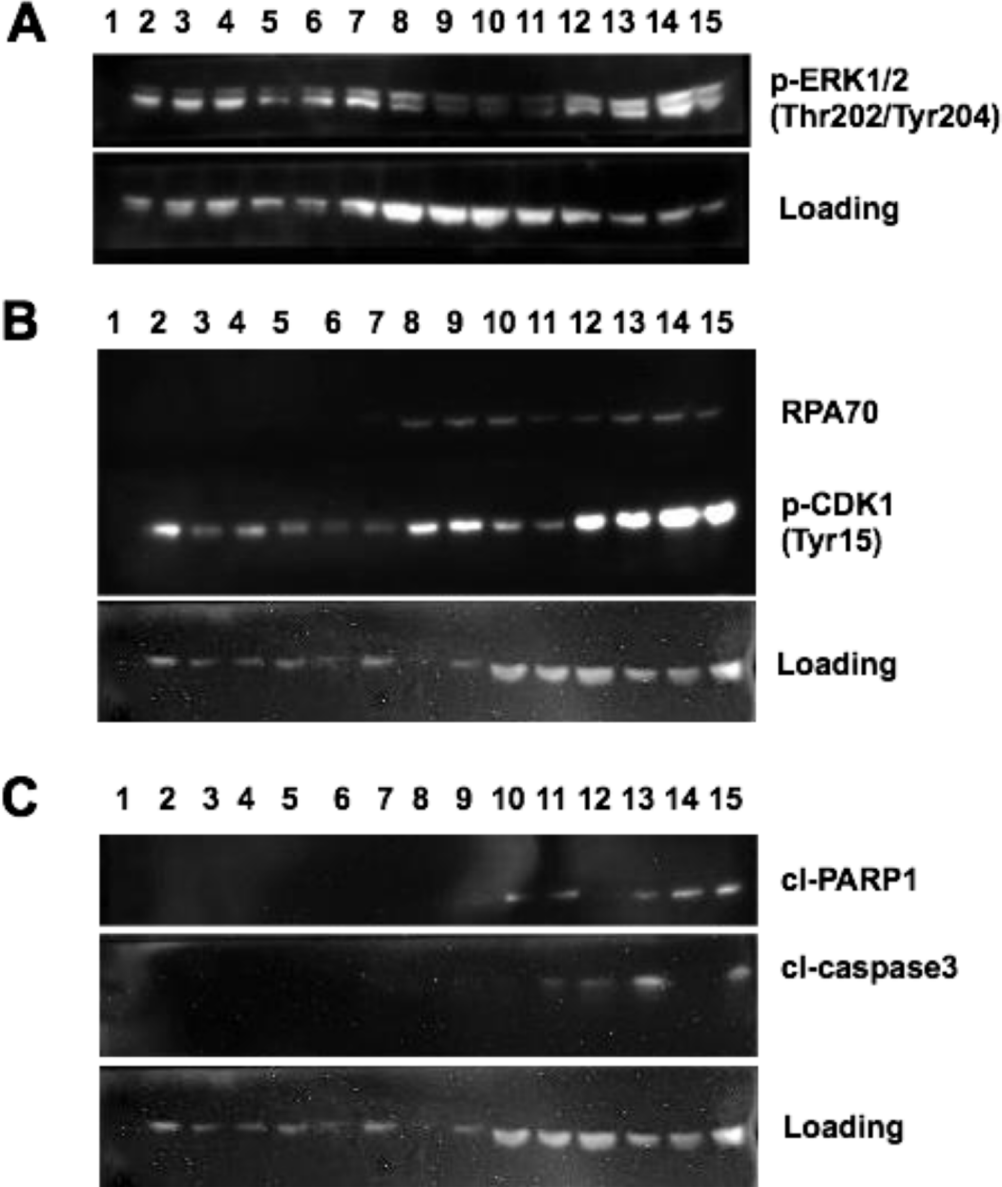
MAPK activation and DNA damage checkpoint induction following MTX + dabrafenib combination treatments (**A**) Western blot of p-ERK1/2 (Thr202/Tyr204) expression in differentially treated SK-MEL-28 and SK-MEL-28VR1 cells. β-actin used as loading control. 30 µg of protein loaded in each lane. (**B**) Western blot of RPA70 and p-CDK1 (Tyr15) expression in differentially treated SK-MEL-28 and SK-MEL28VR1 cells. β-actin used as loading control. 30 µg of protein loaded in each lane. (**C**) Western of cleaved PARP1 and cleaved caspase 3 expression in differentially treated SK-MEL-28 and SK-MEL-28VR1 cells. β-actin used as loading control. 30 µg of protein loaded in each lane.

After confirming MTX induced sensitization of drug-resistant MM cells to dabrafenib, we set out to identify the underlying mechanisms for cell killing induced by the combination treatments. First, we tested for MAPK pathway activation following MTX + dabrafenib, MTX, or dabrafenib treatments. We compared MAPK activation by probing for ERK1/2 threonine 202/tyrosine 204 phosphorylation (p-ERK1/2) (Figure 2A). p-ERK1/2 levels of parental SK-MEL-28 cells remained unchanged following MTX + dabrafenib treatments at 24, 48, and 72-hour time points (Figure 2A, lanes 5–7) when compared to MTX + DMSO (vehicle) treatments at identical time points (Figure 2A, lanes 2–4). However, p-ERK1/2 levels of SK-MEL-28VR1 cells increased following MTX + dabrafenib treatments at 48, 72, and 96-hour time points (Figure 2A, lanes 13–15) compared to MTX + DMSO treatments at identical time points (Figure 2A, lanes 9–11) indicating MAPK pathway induction following MTX + dabrafenib combination treatments.

Next, we investigated MTX-induced DNA damage repair efficiency following dabrafenib treatments. Immunoblotting for the ssDNA binding protein, RPA70 (the 70 kDa DNA binding domain of the replication protein A complex and binds single strand DNA), clearly showed increased expression, which suggests prolonged single strand DNA damage, for 96 hours post dabrafenib treatments compared to DMSO treatments (Figure 2B). SK-MEL-28VR1 cells were simultaneously treated with MTX (75 nM) and either DMSO (Figure 2B, lanes 8–11) or dabrafenib (10 µM) (Figure 2B, lanes 12–15) and harvested at 24, 48, 72, and 96-hour time points. As controls for this experiment, we treated SK-MEL-28 parental cells with an identical treatment scheme as the drug resistant SK-MEL-28VR1 cells (Figure 2B, lanes 2–7). The parental cells did not display RPA70 protein expression 24, 48, or 72 hours following MTX + DMSO (Figure 2B, lanes 2–4) or MTX + dabrafenib (Figure 2B, lanes 5–7) treatments. In contrast, the SK-MEL-28VR1 cells treated with MTX + DMSO exhibited RPA70 expression at 24, 48, and 72-hour time points (lanes 8–10) but not at the 96-hour time point (Figure 2B, lane 11). Interestingly, the SK-MEL-28VR1 cells treated with MTX + dabrafenib expressed RPA70 at the 24, 48, 72, and 96-hour time points (Figure 2B, lanes 12–15). The loss of RPA70 signal in the MTX + DMSO treated SK-MEL-28VR1 cells at 96 hours post MTX treatment suggested the damage was repaired. However, addition of dabrafenib blocked or delayed repair of MTX induced damage even after 96 hours. To our knowledge, this positive correlation between dabrafenib treatment and prolonged ssDNA damage is novel.

Next, we tested whether the observed ssDNA damage was inducing the DNA damage checkpoint in our cells. We therefore monitored tyrosine 15 phosphorylation of Cyclin-dependent kinase 1 (pY15-Cdk1) as a biochemical readout for cell cycle arrest. pY15-Cdk1 is indicative of an active DNA damage checkpoint [36]. The SK-MEL-28VR1 cells exhibited increased phosphorylation of CDK1 at tyrosine 15 following MTX + DMSO treatments at 48 and 72-hour time points (Figure 2B, lanes 9 and 10) compared to parental SK-MEL-28 cells at those time points (Figure 2B, lanes 3 and 4). The presence of pY15-Cdk1 suggested that MTX treatment caused the activation of the DNA damage checkpoint. This comparative trend was accentuated with the MTX + dabrafenib combination treatments. At time points of 24, 48, and 72 hours, SK-MEL-28VR1 cells expressed high levels of pY15-Cdk1 (Figure 2B lanes 12–14) compared to the parental cells at identical time points (Figure 2B, lanes 5–7). Importantly, SK-MEL-28VR1 cells had higher levels of pY15-Cdk1 following MTX + dabrafenib treatments at all time points (Figure 2B, lanes 12–15) compared to MTX + DMSO treatments (Figure 2B, lanes 8–11). These results indicated that the DNA damage checkpoint is active in the SK-MEL-28VR1 cells following damage induction by MTX as indicated by pY15-Cdk1 protein expression. In summary, SK-MEL-28VR1 cells can sustain a cell cycle arrest (based on pY15-Cdk1) yet they cannot repair damage based on of RPA70 expression following dabrafenib treatments. Additionally, the RPA70 and pY15-Cdk1 protein expression levels indicated that the SK-MEL-28VR1 cells are less efficient in repairing MTX-induced single strand DNA damage compared to parental SK-MEL-28 cells, and dabrafenib treatment further compromised MTX induced ssDNA break repair.

Next, we analyzed cell-death induction following the combination treatments. Apoptosis was monitored by detecting cleaved PARP1 [37] and cleaved caspase 3 [38, 39]. The results (Figure 2C) clearly showed increased PARP1 cleavage in SK-MEL-28VR1 cells following MTX + DMSO treatments by 72 hours (lane 10) compared to parental cells at the identical time point (lane 4). PARP cleavage was evident at the 48, 72, and 96 -hour time points (lanes 13–15) in SK-MEL-28VR1 cells following MTX + dabrafenib combination treatments while no PARP cleavage was observed with identical treatments at identical time points in parental cells (lanes 5–7). Cleaved caspase 3 levels confirmed the observed PARP1 cleavage trends. We observed cleaved caspase 3 expression only at the 96-hour time point (lane 11) following MTX + DMSO treatments in SK-MEL-28VR1 cells. In contrast, we observed caspase 3 cleavage by 24 hours following MTX + dabrafenib treatments (lanes 12, 13, and 15). Importantly, we did not observe any caspase 3 cleavage in parental cells under any treatment condition at any time points (lanes 2–7). PARP1 and caspase 3 cleavage patterns in SK-MEL-28VR1 cells treated with MTX + dabrafenib (lanes 12–15) indicated that caspase 3 is cleaved and activated by 24 hours after combination treatments (lane 12), but PARP1 is not cleaved at that time point. In fact, PARP1 is cleaved at the 48-hour time point (lane 13) but cleaved-PARP1 levels at later time points (lanes 14 and 15) were higher compared to the 48-hour time point. Also, the cleaved caspase 3 levels were significantly reduced at 72 hours (lane 14) than at 48 hours and 96 hours. The cleavage patterns of PARP1 and caspase 3 indicated that apoptosis is initiated at 48 hours and then increases at the 72- and 96-hour time points. Collectively, these experiments confirmed the cytotoxicity of MTX + dabrafenib combination treatments in drug resistant MM cells.

### BRAFi resistant MM cells are arrested in G1/S following MTX + dabrafenib combination treatments

**Figure 3:**
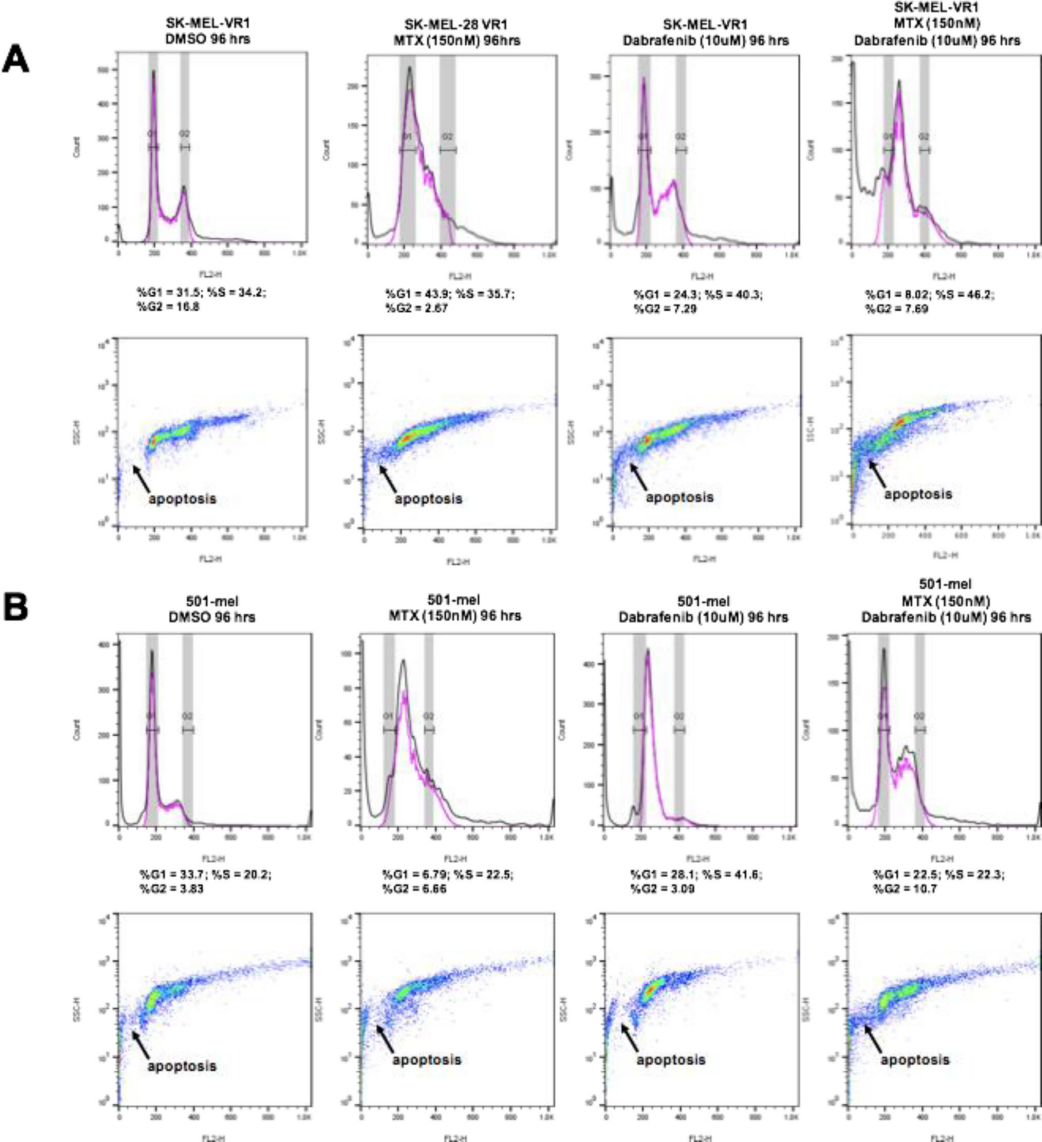
48 hour MTX + dabrafenib treatments cause G1/S cell cycle arrests in BRAFi-resistant MM cells FACScan cell cycle assays following differential treatments of SK-MEL-28VR1 and 501-mel cells. 10,000 cells were analyzed. Top panels are histograms, and bottom panels are corresponding side scatter plots.

Next, we examined the cell cycle profiles of BRAFi resistant MM cells treated with MTX + dabrafenib drug combinations. The cell cycle analysis through FACS was performed on cells treated with 150 nM MTX and 10 µM dabrafenib, alone and in combination, for 96 hours. The results revealed several interesting effects of the drug treatments (Figure 3). In SK-MEL-28VR1 cells (Figure 3A), histograms of DMSO control treatments showed that 31.5% of cells were in G1, 34.2% were in S, and 16.8% were in G2. Following MTX treatments, 43.9% of the cells were in G1, 35.7% were in S, and only 2.67% were in G2. The increase in G1 cell fraction at the expense of the G2 fraction was indicative of cell cycle arrest induction following MTX treatments. Following dabrafenib treatments, 24.3% of cells were in G1, 40.3% of cells were in S, and 7.29% of cells were in G2. This pattern was indicative of S-phase arrest in cells treated with dabrafenib. Importantly, MTX + dabrafenib combination treatments accentuated the S-phase arrest seen with dabrafenib single treatments. Following combination treatments, 8.02% of the cells were in G1, 46.2% were in S, and 7.69% of cells were in G2. Additionally, the side-scatter images of differential treatments in SK-MEL-28VR1 cells (Figure 3A) indicated increased apoptosis with MTX + dabrafenib combination treatments compared to MTX or dabrafenib single treatments. Therefore, taken together the histograms and side-scatter images showed that SK-MEL-28VR1 cells are arrested in S-phase following MTX + dabrafenib combination treatments which ultimately results in cell death induction through apoptosis. Moreover, the FACS data confirmed our immunoblotting observation of increased apoptosis following combination treatments compared to single drug treatments.

The MTX + dabrafenib combination treatments also showed interesting trends in 501-mel cells (Figure 3B). These cells were shown to be intrinsically resistant to dabrafenib in colony formation and spheroidal growth assays but sensitive to the MTX + dabrafenib combination treatments (Figures 1B and 1C). Cell cycle analysis following 96-hour MTX + dabrafenib treatments revealed that 22.5% of 501-mel cells were in G1, 22.3% were in S, and 10.7% were in G2 (Figure 3B; histograms). In comparison, DMSO treatments showed that 33.7% of the cells were in G1, 20.2% were in S, and 3.83% were in G2. Although the cell cycle percentage numbers were similar for both treatments, the side-scatter images (Figure 3B) showed an increase in apoptosis following combination treatments compared to DMSO treatments. MTX single treatments arrested 501-mel cells in S-phase (Figure 3B; histograms). 6.79% of cells were in G1, 22.5% were in S, and 6.66% were in G2. Following dabrafenib single treatments, 28.1% of 501-mel cells were in G1, 41.6% were in S, and only 3.09% were in G2 indicating a G1/S arrest. Importantly, the side-scatter images (Figure 3B) clearly showed increased apoptosis in 501-mel cells treated with the combination treatments compared to MTX or dabrafenib single treatments. Collectively, the SK-MEL-28VR1 and 501-mel cell cycle analysis showed that cells were arresting as a result of the combination treatments, and increased apoptosis was observed following combination treatments compared to single MTX, dabrafenib, or DMSO treatments. Thus, the FACS analysis confirmed trends observed from immunoblotting.

### BRAFi acquired resistant SK-MEL-28VR1 cells have similar activating mutations in RAS as 501-mel cells

Next, we analyzed the mutational profiles of the acquired-resistant SK-MEL-28VR1 cells and parental SK-MEL-28 cells to the known mutational profiles of intrinsically-resistant 501-mel cells (canSAR 3.0) to potentially correlate MTX + dabrafenib cell sensitivity to specific mutational patterns. RNAseq analysis confirmed that parental SK-MEL-28 cells expressed *BRAF* V600E; however, the SK-MEL-28VR1 cells were revealed to be WT for BRAF (Table 1). Next, we examined TP53 mutations since we observed a G1 delay from our FACS analysis and WT TP53 activity is known to be critical for G1 arrests in cancer cells following treatments with DNA damaging agents [40]. We confirmed that SK-MEL-28 parental cells harbored L145R TP53 mutations (Table 1). The SK-MEL-28VR1 cells harbored multiple TP53 mutations (Table 1). 501-mel cells have WT TP53 (CanSAR 3.0). Therefore, TP53 mutational status did not correlate with G1 delays observed from our cell cycle assays. Examining other genes in the MAPK pathway, we discovered that the SK-MEL-28VR1 cells harbored homozygous G12V activating KRAS mutations (Table 1). We confirmed that our parental SK-MEL-28 cells were KRAS WT. KRAS is a GTPase of the RAS family which lies directly upstream of BRAF in the MAPK cascade [41]. Interestingly, *BRAF* V600E 501-mel cells are known to harbor G12D activating mutations in the NRAS gene (canSAR 3.0). KRAS, NRAS, and HRAS are the 3 members of the RAS family of GTPases that are the most prevalent oncogenes in cancer progression and have similar cellular functions [42].

**Table 1:** Mutational status of MM cells

Cell lines	Genes	mutational status	BRAF sensitivity
SK-MEL-28	BRAF	V600E	sensitive
	RAS	WT	
	CTNNB1	splice donor variant	
	CSDE1	splice donor variant	
	TP53	L145R	
SK-MEL-28VR1	BRAF	WT	resistant
	RAS	KRAS G12V	
	CTNNB1	splice donor variant	
	CSDE1	splice donor variant	
	TP53	P72R, R273H, P309S	
MEL501	BRAF	V600E	resistant
	RAS	NRAS G12D	
	CTNNB1	S37F, D32H	
	CSDE1	G12D	
	TP53	WT	

A total of 5 genes (BRAF, NRAS, CTNNB1, and CSDE1) have previously been reported to be mutated in the 501-mel (canSAR 3.0). We examined the CTNNB1 and CSDE1 genes for mutations in the SK-MEL-28VR1 line and the parental line. Both SK-MEL-28VR1 and the parental cells exclusively expressed previously identified splice donor variants of CTNNB1 and CSDE1 (Table 1). Although we did not analyze genomic data, the RNAseq data show that mutations that altered the splicing pattern must have occurred in both cell lines. For both genes, >90% of all mRNAs sequenced of these genes are the splice donor variants and not the full-length mRNA which indicates that the splice donor variant is the major proportion of transcriptional products for these genes and essentially tell us that the gene products are rendered non-functional.

Splice donor variants have mutations in the 2 base regions at 5’ ends of introns and result in alternative splicing that can disrupt normal gene expression [43]. Overall, transcriptome analysis revealed similar mutational profiles between the BRAFi acquired-resistant (SK-MEL-28VR1), intrinsically-resistant (501-mel), and sensitive parental SK-MEL-28 cells with the exceptions being the BRAF and RAS genes. The sensitive SK-MEL-28 are *BRAF* V600E; KRAS WT, the intrinsically-resistant 501-mel cells are *BRAF* V600E; NRAS G12D, and the acquired-resistant SK-MEL-28VR1 cells are BRAF WT; KRAS G12V. Since SK-MEL-28VR1 and 501-mel cells were both mutated at codon 12 of *RAS* genes while the sensitive SK-MEL-28 cells were RAS WT. We postulate that the activating RAS codon 12 mutation exerts dominance over BRAF mutations and determines resistance to MTX + BRAF inhibitors in MM cells. Additionally, the G1 delay observed with combination treatments in our cell cycle assays (Figures 3B and 3C) seem to be independent of TP53 mutational status (Table 1).

## DISCUSSION

BRAFi acquired resistance is a persistent problem in MM therapy even when given in combination with MEK inhibitors [44]. High rates of tumor relapse contribute to a low 5-year survival rate in MM. In this study, we have identified a novel combination treatment scheme that is cytotoxic to drug resistant MM cells. MTX is a folate analog that does not have activity against MM as a single agent. We show that when used in combination, MTX sensitizes BRAFi resistant MM cells to dabrafenib. We describe a novel positive correlation between dabrafenib and ssDNA break repair delay. Additionally, we show that BRAFi resistant MM cells are arrested following MTX + dabrafenib combination treatments by 96 hours. Importantly, we elucidate the induction of Caspase 3 activated apoptotic cell death during this arrest. Finally, we identify RAS activating mutations at codon 12 that may predict the efficacy of MTX + dabrafenib combination treatments.

BRAFi acquired-resistant (SK-MEL-28VR1) and intrinsically-resistant (501-mel) MM cells were resistant to dabrafenib in 2D colony formation (Figures 1A and 1B) and 3D spheroidal (Figure 1C) assays. We expected the SK-MEL-28VR1 cells to be dabrafenib resistant since these cells were initially identified as vemurafenib resistant clones of the parental SK-MEL-28 MM line. Vemurafenib is a BRAFi that exerts similar effects on the MAPK pathway as dabrafenib [45]. Similarly, 501-mel has been shown to be resistant to BRAFi’s despite harboring *BRAF* V600E mutations [46]. Previously, we had identified the serine synthesis pathway as a critical determinant of BRAFi resistance in MM cells [1]. Moreover, in that study we observed inductions in cell proliferation of SK-MEL-28VR1 cells following BRAFi treatments. Therefore, we postulated that serine synthesis pathway induction contributed to the higher nucleotide and amino acid production necessary to support higher rates of cell proliferation. Since serine synthesis lies directly upstream and contributes precursors to the folate cycle which feeds into the nucleotide synthetic pathways, we hypothesized that folate cycle inhibitors (antifolates) may enhance the sensitivity of BRAFi’s in resistant MM cells. In this study, we confirmed our hypothesis and identified MTX as a novel sensitizer of BRAFi resistant cells to dabrafenib (Figure 1A–1C). Moreover, we showed in spheroidal growth assays (Figure 1D) that MTX sensitizes MM cells to encorafenib, another BRAFi that is known to cause senescence and autophagy. The MTX + encorafenib experiments displayed that increased efficacy in combination with MTX was a feature general to BRAFi’s and not specific to dabrafenib.

Additionally, we identified a novel dabrafenib induced DNA damage repair delay in BRAFi resistant MM cells. Through immunoblotting, we showed that the SK-MEL-28VR1 cells were less efficient in DNA damage repair than the BRAFi sensitive parental SK-MEL-28 cells following MTX treatments (Figure 2B). Importantly, we showed that MTX induced single strand breaks were prolonged in SK-MEL-28VR1 cells following dabrafenib treatments compared to control DMSO treatments (Figure 2B). We examined RPA70 levels to identify single strand DNA breaks. RPA70 is the DNA binding domain of the RPA complex, the standard sensor of single strand breaks in human cells. We also assessed phosphorylation of Cdk1 at tyrosine 15 to identify active DNA damage checkpoints in our cells. This phosphorylation is indicative of an active cellular DNA damage checkpoint and cell cycle arrest [47]. Immunoblots showed a prolonged active DNA damage checkpoint following MTX + dabrafenib treatments even 96-hours post treatment (Figure 2B). Next, we confirmed the cytotoxicity of the combination treatments. Immunoblots clearly identified apoptotic cell death induction via PARP1 and caspase 3 cleavage in SK-MEL-28VR1 cells following MTX treatments while no cell death induction was observed in the parental SK-MEL-28 cells (Figure 2C). The onset of cell death was faster in MTX + dabrafenib treated cells versus MTX + DMSO treated cells. Collectively, these experiments identify a novel connection between dabrafenib and repair delay of MTX induced ssDNA breaks and confirm the cytotoxicity of MTX + dabrafenib combination treatments in BRAFi resistant MM cells.

Next, we utilized FACS cell cycle analysis to identify a G1/S delay following combination treatments in SK-MEL-28VR1 (Figure 3A) and 501-mel cells (Figure 3B). Single MTX, dabrafenib, and MTX + dabrafenib treatments caused G1/S arrests in the BRAFi resistant cell lines. However, apoptotic cell fractions increased in both cell lines with the combination compared to either single drug treatments (Figures 3A and 3B, side scatter plots). This data clearly showed that although single MTX and dabrafenib single treatments seem to be cytostatic to BRAFi resistant cells, MTX + dabrafenib treatments induce apoptotic cell death making the combination cytotoxic. Overall, immunoblotting and cell cycle analysis revealed that MTX + dabrafenib treatments were causing G1/S arrests via ssDNA break induction by MTX coupled with dabrafenib induced DNA damage repair delay. Ultimately, the prolonged cell cycle arrest with unrepaired DNA triggered apoptotic cell death.

Lastly, we compared mutational profiles of BRAFi resistant SK-MEL-28VR1 and 501-mel cells and identified similar codon 12 activating mutations in RAS genes (Table 1). Transcriptomic profiling elucidated WT BRAF expression in SK-MEL-28VR1 cells while the parental SK-MEL-28 cells were confirmed to be *BRAF* V600E. However, the parental cells were RAS WT while the BRAFi acquired-resistant cells were *KRAS* G12V. 501-mel cells are known to be G12D for *NRAS, BRAF V600E*, and BRAFi resistant. Thus, we believe the RAS activating G12 mutations are critical for MTX + dabrafenib combination therapy efficacy in MM. Since RAS is shown to exert dominance over downstream MAPK members in activating the pathway in cancer cells [48], and RAS mutations are known to accentuate the allosteric MAPK activating effects of BRAFi’s in BRAF WT MM cells [49], activating codon 12 RAS mutations may potentially be biomarkers of efficacy for MTX + dabrafenib combination treatments independent of BRAF mutational status. Examining our immunoblotting, cell cycle, and mutational profiling data collectively, we hypothesize that nucleotide pool depletion by competing cell signals of MTX induced ssDNA damage repair and dabrafenib induced MAPK pathway activation ultimately triggers cell death in BRAFi resistant MM cells. We are currently performing experiments to test this hypothesis.

In totality, our experiments have elucidated a novel positive correlation between dabrafenib treatments and prolonged MTX induced ssDNA breaks in BRAFi resistant MM cells. To our knowledge, these studies are the first to connect dabrafenib or any other BRAFi’s to prolonged DNA breaks. We believe cells with prolonged G1/S arrests induced by ssDNA break repair delays following MTX + dabrafenib treatments ultimately die through apoptosis. We exploit this novel phenomenon by sensitizing BRAFi acquired- and intrinsically-resistant MM cells to dabrafenib via simultaneous MTX treatments. We postulate that nucleotide pool depletion and ssDNA break induction by MTX may disrupt downstream transcriptional reprogramming activated by the dabrafenib induced MAPK pathway ultimately triggering cell death. Excitingly, we have potentially identified a mutational biomarker in the well-known 12^th^ codon of RAS as a determinant of efficacy of our MTX + dabrafenib combination treatments. Ongoing *in vitro* and *in vivo* experiments are being performed to further confirm this potentially important genetic trend. The MTX + dabrafenib (or other BRAFi’s such as vemurafenib or encorafenib (LGX818)) combination therapy has the potential to positively impact patient survival following MM relapse, and our ongoing and future experiments are designed to accelerate combination treatments along translational pipelines. We have previously reported on the efficacy of DNA damagers + BRAFi treatments in multiple cancer cell types including pancreatic and non-small cell lung cancers [1] and believe that the identified method of cell death induction highlighted in this study can potentially be a novel, broad-reaching method of cell death induction common to RAS hyperactivated cancer cells which constitute over 16% of all patient profiles across all cancer types in the clinic (AACR Project Genie, 2017).

## MATERIALS AND METHODS

### Cell culture and chemicals

SK-MEL-28 and 501-mel cells were a gift from Dr. Alfonso Bellacosa at Fox Chase Cancer Center (FCCC). SK-MEL-28 and 501-mel cells were authenticated by the FCCC cell culture core according to ATCC test recommendations. The SK-MEL-28VR1 cell line was identified through progressive vemurafenib selection as previously described [1]. All cell lines were reanimated less than 6 months before experimentation. Cell lines were cultured in RPMI1640/10%FBS (GenDepot) supplemented with 2mM glutamine (Life Technologies; 25030081) and were maintained at 37C in 5% CO2. Methotrexate, dabrafenib, and encorafenib were obtained from Selleckchem.

### Cell viability assays

2D Colony formation assays were plated as previously described [1]. Cells were treated with DMSO, MTX, dabrafenib, or MTX + dabrafenib at various doses on day 1 for 48 hours. Day 4, drugs added on day 1 were washed out. Cells were allowed to grow for 7 days and fixed (10% methanol + 10% acetic acid) and stained with crystal violet (0.4% in 20% ethanol) for quantitation at 595 nm. 3D spheroidal assays were plated as previously described [1]. Cells were plated in 96-well spheroid plates (Corning CLS4515) according to cell line-specific plating efficiencies that allowed for >500 μm in diameter of spheroid growth after 48 hours. Cells were treated with DMSO, MTX, darafenib, encorafenib, MTX + dabrafenib, or MTX + encorafenib at various doses on day 2 for 96 hours. Cell growth was subsequently analyzed using Cell Titer Glo 3D (Promega).

### Immunoblotting

Cells were harvested and lysed in buffer (1% NP40/PBS/10% glycerol) with protease and phosphatase inhibitors. Protein concentrations were determined with Total-Protein-Assay-kit (ITSI Biosciences; K-0014-20) and then SDS sample buffer was added to the lysates. 50µg of boiled lysates were separated by SDS-PAGE and transferred onto PVDF membranes (G-Biosciences; 786-018PV). p-ERK1/2 (4370), p-CDK1 (4539), RPA70 (2267), cl-PARP1 (5625), and cl-caspase 3 (9664) primary antibodies were obtained from Cell Signaling Technologies (CST). β-actin (CST 8457) primary antibodies were used as loading controls. Anti-rabbit IgG, HRP-linked antibody (CST 7074) was used as the secondary. FemptoLUCENT Plus HRP Kit (G Biosciences; 786-003) was used as the substrate for visualization.

### Fluorescence-activated cell sorting (FACS) analysis

SK-MEL-28, SK-MEL-28VR1, or 501-mel cells were plated in 10cm dishes and allowed to grow to a confluence of 60%. Subsequently, cells were treated with differential drug treatments. Cells were either treated with DMSO, MTX (75 nM), dabrafenib (10 µM), or MTX (75 nM) + dabrafenib (10 µM) treatments. Following 48 hours of drug exposure, cells were trypsinized and harvested. Cells were pelleted and washed in PBS before being fixed in cold 70% ethanol. Fixed cells were stained with Propidium Iodide (PI) (5 µg/ml) for 30 minutes at 37° C in the dark. Cell cycle was analysed by flow cytometry, using a FACScan Flow analyzer (BD Biosciences) operated by CellQuest software, and 10,000 events were collected per sample. Data was analyzed using FlowJo software (Version 10). Forward and side-scatter profiles were obtained from all samples.

### RNA sequencing and transcriptomic analysis

10 million cells were pelleted and sent to Quick Biology (Pasadena, CA) for RNA extraction and sequencing (RNA-Seq). Libraries for RNA-Seq were prepared with KAPA Stranded RNA-Seq Kit. The workflow consists of mRNA enrichment, cDNA generation, and end repair to generate blunt ends, A-tailing, adaptor ligation and PCR amplification. Different adaptors were used for multiplexing samples in one lane. Sequencing was performed on Illumina Hiseq3000/4000 for a pair end 150 run. Data quality check was done on Illumina SAV. Demultiplexing was performed with Illumina Bcl2fastq2 v 2.17 program.

The raw RNA sequencing read files were pre-processed using Cutadapt [26] to remove adapter sequences and poly-A tails. Next, fastQC (quality control) calculations were used to confirm elimination of over-represented sequences, as well as to provide additional QC metrics. The pre-processed fastq files were then aligned to GRCh38 reference using STAR [27]. STAR was also subsequently used to sort and mark duplicate reads in aligned bam files. GATK [28] was used to perform a Split N’ Trim operation for all spliced reads within bam files, which were then indexed using SAMtools [29]. The resulting finished bam files were then used as the input to both variant calling and RNA expression quantitation. The pool of finished bam files from all replicates was used to perform germline haplotype variant calling using FreeBayes [30]. Variant calls produced include insertions, deletions, as well as single-nucleotide and complex polymorphisms. The resulting raw variant panel was filtered to retain variants based on a minimum read depth of 4, and minimum quality of Phred 30. Boolean operations on raw variant panels from multiple samples are computed using VCFTools [31].

The web-based tool gene.iobio.io was used to graphically explore VCF files by providing a list of genes to analyze. Internally, it uses Ensembl’s VEP [32] program to provide estimates of variant effect (missense, frameshift, stop gain/loss, splice modifier), pathogenicity, and supporting clinical data. Cufflinks [33, 34] was used to perform transcript quantitation and normalization from the finished bam file of each replicate. A mask was specified to exclude transcripts belonging to rRNA, tRNA, mtRNA genes. Cuffmerge was used to combine the replicates of each sample into a master transcriptome assembly, from which Cuffquant computed the gene and transcript expression profiles. Cuffnorm used these profiles to provide normalized expression levels that could be compared between samples. When two or more samples were included in the Cuffquant expression profile computation, Cuffdiff [35] was used to perform pair-wise differential expression analysis between the included samples.

## Abbreviations

MM: metastatic melanoma
MAPK: mitogen-activated protein kinase
BRAF: v-Raf murine sarcoma viral oncogene homolog B
BRAF V600E inhibitors: BRAFi’s
RPA70: 70 kDa DNA binding domain of the replication protein A complex
pY15-Cdk1: Tyrosine 15 phosphorylated cyclin dependent kinase 1
PARP1: Poly [ADP-ribose] polymerase 1
ERK1/2: Mitogen-activated protein kinases, extracellular signal–regulated kinases
